# A Vulnerability Assessment of 300 Species in Florida: Threats from Sea Level Rise, Land Use, and Climate Change

**DOI:** 10.1371/journal.pone.0080658

**Published:** 2013-11-19

**Authors:** Joshua Steven Reece, Reed F. Noss, Jon Oetting, Tom Hoctor, Michael Volk

**Affiliations:** 1 Department of Biology, Valdosta State University, Valdosta, Georgia, United States of America; 2 Department of Biology, University of Central Florida, Orlando, Florida, United States of America; 3 Florida Natural Areas Inventory, Florida State University, Tallahassee, Florida, United States of America; 4 Center for Landscape Conservation Planning, University of Florida, Gainesville, Florida, United States of America; University of Saskatchewan, Canada

## Abstract

Species face many threats, including accelerated climate change, sea level rise, and conversion and degradation of habitat from human land uses. Vulnerability assessments and prioritization protocols have been proposed to assess these threats, often in combination with information such as species rarity; ecological, evolutionary or economic value; and likelihood of success. Nevertheless, few vulnerability assessments or prioritization protocols simultaneously account for multiple threats or conservation values. We applied a novel vulnerability assessment tool, the Standardized Index of Vulnerability and Value, to assess the conservation priority of 300 species of plants and animals in Florida given projections of climate change, human land-use patterns, and sea level rise by the year 2100. We account for multiple sources of uncertainty and prioritize species under five different systems of value, ranging from a primary emphasis on vulnerability to threats to an emphasis on metrics of conservation value such as phylogenetic distinctiveness. Our results reveal remarkable consistency in the prioritization of species across different conservation value systems. Species of high priority include the Miami blue butterfly (*Cyclargus thomasi bethunebakeri*), Key tree cactus (*Pilosocereus robinii*), Florida duskywing butterfly (*Ephyriades brunnea floridensis*), and Key deer (*Odocoileus virginianus clavium*). We also identify sources of uncertainty and the types of life history information consistently missing across taxonomic groups. This study characterizes the vulnerabilities to major threats of a broad swath of Florida’s biodiversity and provides a system for prioritizing conservation efforts that is quantitative, flexible, and free from hidden value judgments.

## Introduction

The combination of accelerated climate change, habitat loss and degradation, invasive species, overharvesting, sea level rise and other threats have dramatically increased extinction rates during recent centuries [1-3]. Many refer to this phenomenon as the Biodiversity Crisis [4-7]. Conservation efforts to mitigate threats and preserve biodiversity consistently fall short. Whereas much of this failure can be attributed to insufficient funding for conservation [8-10], efforts are underway to improve the cost-efficiency of conservation actions [11-15]. Prioritization systems are often used to target funds toward the biologically richest or most imperiled regions around the globe [16,17], or to the most threatened and valued species within a region [18-21]. Traditionally, tools such as the Conservation Status Assessment (i.e., NatureServe’s Global/National/State ranking system for species and natural communities), the US Endangered Species Act, and the IUCN Red List are implemented to prioritize conservation efforts based on rarity, threats, or patterns of decline. More recently, threats from climate change have been incorporated into prioritization systems [16,22-25]; however, few systems explicitly account for threats related to sea level rise (SLR), even though it is directly related to climate change. In addition to threats from SLR and climate (temperature and precipitation) change, coastal species face growing pressure from human populations, approximately half of which live within 200 kilometers of a coastline [26]. Therefore, prioritization schemes implemented in coastal regions should jointly consider threats to biodiversity from SLR, climate change, and human land use. 

Florida is a hotspot of endemism for plants [27,28], vertebrates [29,30], and insects [31] outside of the tropics. This biodiversity is threatened by increasing urbanization; Florida is the fourth most populous state in the US and the third fastest growing state (US Census 2010), with more than 17% net increase in population from 2000 to 2010. Land use conversion from natural areas to farm, pastureland, or urban areas is rapid, and by 2060 an additional 2.7 million acres of undeveloped and agricultural lands are projected to be converted to urban areas to accommodate population growth [32]. Florida is also highly threatened by SLR, storm surge, and salt water intrusion; the state has approximately 1200 miles of coastline, with the maximum distance from the coast less than 150 km. Coastal erosion due to increasingly strong hurricanes and storm surge combined with SLR and coastal armoring create a unique suite of interacting threats to Florida's biodiversity [33]. Given these synergistic threats and meager conservation resources, conservation efforts should target species and assemblages that are most highly imperiled and of greatest ecological, evolutionary, or other value, while also being feasible to save (salvageable). 

In this paper we describe a two-step method for prioritizing conservation efforts for species threatened by climate change, SLR, and land-use change projected by the year 2100. Our methods are complementary to existing prioritization schemes because we use most of the same criteria, but in addition we explicitly account for the effects of sea level rise in addition to factors traditionally considered in prioritization schemes. First, we compiled a list of 300 species identified by the Florida Natural Areas Inventory (FNAI) and the Florida Fish and Wildlife Conservation Commission as vulnerable to extinction in Florida or globally due to SLR and other threats. Second, we applied a novel vulnerability assessment, the Standardized Index of Vulnerability and Value (SIVVA) [34], which uses both empirical estimates and expert opinion to assess species’ joint vulnerability to climate change, sea level rise, land-use change, and other threats, but also ranks species by their ecological and other conservation values. This work identifies species in Florida that warrant immediate attention in conservation planning and presents a new approach to prioritizing conservation efforts for those species.

## Materials and Methods

### Candidate Species for Vulnerability Assessment

The Florida Natural Areas Inventory (FNAI) tracks approximately 1000 species of plants and animals of conservation concern in Florida. For each species FNAI keeps a spatially explicit database on distribution in the form of “element occurrences,” i.e., points or polygons representing observed species localities. Element occurrences correspond to discrete populations based on recorded or estimated dispersal and connectivity. Thus, species with state-wide distributions and high levels of connectivity may have a few large element occurrences throughout the state, while other species that are spottily distributed throughout the state and lacking connectivity may have several smaller and discrete element occurrences. Available funding and staff limited our ability to conduct vulnerability assessments for all 1000+ species. All species tracked by FNAI are vulnerable to extinction, but we wanted to identify those most at risk from SLR in addition to other threats. To do this, we used a 10 m digital elevation model (DEM) to create a “bathtub” projection showing 2 m of SLR. We limited our search to species for which some element occurrences overlapped with this SLR projection, assuming that this is a relatively liberal projection for the year 2100. Next, we identified species for which 50% of their element occurrences were each inundated by 50% or more according to our bathtub model. An additional 32 species of conservation concern were added to this list to conform to requirements of one of our funding agencies, which identified these species as of high conservation concern. 

### Executing the SIVVA Survey

To continue our species prioritization, we developed an expert opinion-based survey called the Standardized Index of Vulnerability and Value Assessment (SIVVA). Previously, we showed that existing vulnerability assessments and species prioritization protocols inconsistently estimated the vulnerability of species in Florida [34] and failed to adequately characterize the multiple threats facing Florida’s biodiversity. Some of this variation was related to assessments of different taxonomic groups or at varying spatial scales, but much of the variation reflected differences in assessment criteria. We developed SIVVA to address these shortcomings by standardizing multiple disparate vulnerability assessments and combining information on different types of threats with other metrics important to prioritization such as ecological, evolutionary, cultural, or economic value. Thirty criteria are distributed across four modules in SIVVA: 1) Vulnerability, 2) Lack of Adaptive Capacity, 3) Conservation Value, and 4) Information Availability. The tool is described in detail, and a list of all of the criteria in each module are given in Reece and Noss [34]. Additional information on individual criteria and instructions provided to assessors can be viewed in an example SIVVA evaluation spreadsheet (http://noss.cos.ucf.edu/publications/sivva) and in Table S1. We use the term “vulnerability” to refer to the combination of exposure and sensitivity to threats, and we consider the adaptive capacity of populations separately from vulnerability (although future users could treat these as a combined module). Because the final SIVVA score included metrics of conservation value and information availability, we refer to the combined SIVVA evaluation as “priority.” 

For each of the 300 species identified in Table S2, we solicited experts who authored papers or conducted studies on the species or were directly involved in their management. All experts were provided with a summary and bibliography of the available literature on the species. Despite the drawbacks sometimes associated with expert-opinion based assessments [35,36], expert judgment in combination with published literature has been shown to be highly accurate [37], especially when accounting for expert uncertainty [38]. Experts were given SIVVA in the form of an Excel Worksheet, maps of projections of 0.5, 1.0, 2.0, and 3.0m of SLR [39-41], projected changes in temperature and precipitation (see below), projections of land-use change, and a summary of the literature available on the species. We asked assessors to evaluate the impacts of future climate and SLR based on these detailed projections. 

To project climate impacts, we used the Nature Conservancy Climate Wizard (www.climatewizard.org) and statistically downscaled global projections for a ‘medium’ (A1B) Emission Scenario (ES), and an Ensemble Average General Circulation Model (GCM) following the IPCC Fourth Assessment. We calculated the change in mean annual temperature in Florida from data modeled from 1900 to 2000 relative to temperature projections modeled from 2000 to 2100. We compared mean annual precipitation under the same GCM and ES above from modeled 1900-2000 and modeled 2000-2100 data. We calculated the difference between wet (June, July, and August) and dry season (December, January, and February) rainfall modeled over 1900 to 2000, and compared that to the difference between wet and dry season rainfall modeled over 2000 to 2100. This difference describes seasonal variability in rainfall, irrespective of total annual rainfall. We assessed land-use change using the projections provided in the Florida 2060 report [42], the only statewide projection of population growth and land-use conversion available at the time of this study. 

Similar to other vulnerability assessments, we asked experts to rank species on a scale from 1 to 6 for each of 30 criteria (Table S1), where a score of zero means that insufficient information exists to assess that criterion, a score of 3 corresponds to no effect, scores of 4, 5, and 6 correspond to increasingly negative effects, and scores of 2 and 1 correspond to increasingly positive effects. In addition to the scores, each criterion was given a weight that corresponds to our estimation of its relative importance (Table S1); these weights were randomized in subsequent analyses to identify the impact of our weighting scheme on overall prioritizations (see below). A summary score was computed for each module as the total number of points (weight of the criteria multiplied by the score from 1 to 6) divided by the total possible number of points if each criterion scored had received the maximum score. Thus, all SIVVA scores can be presented on a scale from zero to one corresponding to minimum and maximum priority.

### SIVVA Quality Control-Agreement in Expert Opinion

A subset of 40 species was assessed by at least two experts. To assess biases among experts we conducted an analysis of variance (ANOVA) for each of the four modules to determine if a significant portion of the variation in final scores was explained by variation among expert assessors. We reconciled two independent valuations of each species by first testing if the difference between the two assessors for the final score of each module was less than 95% of the distribution of pairwise differences among all other species. This approach is based on the expectation that variation among assessors is less than variation among species. We then reported the average score of the two valuations. For all remaining assessments, we used the evaluations of a single expert or a single group of experts and review by the first author to ensure compliance with SIVVA design and that adequate justification existed for their assessments of each criterion. The vast majority of assessments were completed in the presence of or over the phone with the authors of SIVVA.

### Accounting for Uncertainty

This assessment addressed three types of uncertainty: 1) expert uncertainty – when an expert believes that more than one value is equally likely to represent the true value for a criterion; 2) insufficient knowledge – when a small number of criteria are assessed due to limited knowledge about the species; 3) weighting uncertainty – when one or two criteria contribute disproportionately to the priority score for a species. Some VAs, such as the Climate Change Vulnerability Index (CCVI) [23,24], account for expert scoring uncertainty, but most ignore the latter two types of uncertainty. We account for expert scoring uncertainty with a check-box next to each criterion, where experts can note if they are not sure of the proper score. When the uncertainty box was checked, we quantified scoring uncertainty in the final computing of scores by adding 0, +1, or -1 to each criterion's score and recalculating the effect on the overall score using 1000 Monte Carlo simulations. We account for knowledge uncertainty by reporting on the proportion of criteria scored. Finally, we assess weighting uncertainty through 1000 Monte Carlo simulations where criterion weights are randomly drawn from the set of user-defined weights (in our example, weights are 0.25, 0.5, 1, 2, and 4). As a summary statistic, we report on the combined uncertainty of the three types described above for each module by taxonomic group. Combined uncertainty was computed as the maximum and minimum values across all types of uncertainty (i.e., not compounding uncertainty). 

### SIVVA Reporting

Unless indicated otherwise, SIVVA results are reported based on scores averaged across all four SLR scenarios. Data are available on each of those SLR scenarios individually, but for simplicity we present here the averages across all scenarios. First, we identify those species for which extinction appears highly probable by 2100. Second, we report uncertainty measures and identify types of missing data by taxonomic group. Third, we report on the range of values suggested by experts across all modules to broadly characterize vulnerabilities, adaptive capacities, conservation values, and information availability. Fourth, we describe taxonomic patterns of vulnerability, and differences between listed and non-listed taxa at the State and Federal level, by comparing their SIVVA scores using t-tests assuming unequal variance. Fifth, we prioritize species based on SIVVA under five approaches that each emphasizes different types of information. Because all prioritization schemes contain bias, for example taxonomic or societal biases, we chose a variety of approaches, which is a strength of the SIVVA framework. We are unaware of similar flexibility in other existing assessments. The following are five prioritization schemes implemented within SIVVA to reflect differences in conservation priorities.

1 Stepwise Prioritization: We identified from our list of 300 species those that were above a threshold of Conservation Value by identifying natural breaks in the distribution of Conservation Value scores. We detected a natural break by ranking species from high to low by their Conservation Value scores, which resulted in a logistic curve. The mean and median values were identical, and were used as the cutoff. From these species we prioritized those with the highest scores for the combination of Vulnerability and Lack of Adaptive Capacity, where each module was weighted by the number of criteria (12 Vulnerability criteria versus 6 for Lack of Adaptive Capacity). We examined Information Availability scores to identify the types of data gaps critical to fill for the species at highest risk of extinction.
2 Equal Weighting: Each of the four SIVVA modules contributed equally to the final scores; criteria within each module were weighted as shown in Reece and Noss [34].
3 Emphasis on Vulnerability: The overall rank or score for each species is the weighted average of scores across all four modules, where Vulnerability (45%) and Lack of Adaptive Capacity (25%) together make up 70% of the final score, Conservation Value contributes an additional 20%, and Information Availability contributes the final 10%.
4 Emphasis on Conservation Value: Conservation Value contributed 50% of the final score, with 20% from Vulnerability, 20% from Lack of Adaptive Capacity, and 10% from Information Availability.
5 Emphasis on Conservation Value and Information Availability: Vulnerability and Lack of Adaptive Capacity each contributes 15%, and Conservation Value and Information Availability each contribute 35% towards the final score.

## Results

At the time of this study the FNAI tracking list consisted of 1049 species, of which 519 had distributions that overlapped our bathtub inundation model based on 2m of SLR. From this list, 268 species were projected to have 50% or more of their element occurrences inundated by at least 50% (by area). The additional 32 species of conservation concern resulted in a total of 300 species to be evaluated by SIVVA (Table S2). The taxonomic breakdown of this list was approximately 39% plants, 22% invertebrates, 15% birds, 10% mammals, 8% reptiles, 5% fishes, and 2% amphibians. The low number of amphibians in our study reflects the concentration of rare amphibians primarily in the northern panhandle of Florida, well inland of potential inundation from SLR. FNAI only tracks 16 species of amphibians and few of these are significantly threatened by SLR, which was a major focus of our study. Of these 300 species, 120 are listed as Endangered (28 under the US ESA and 92 under the FL ESA) and 35 are Threatened (15 US ESA and 20 FL ESA). Results of climate (temperature and precipitation), land-use, and SLR projections can be viewed in Reece and Noss [34] and at http://noss.cos.ucf.edu/publications/sivva.

### SIVVA Quality Control-Agreement in Expert Opinion

Expert assessments for the 40 species evaluated independently by multiple experts were remarkably similar, which largely reflects a carefully crafted assessment scale [34]. None of the ANOVA comparisons revealed a significant effect of assessor on the SIVVA score for Vulnerability (F = 0.12, P = 0.73), Lack of Adaptive Capacity (F = 0.01, P = 0.93), Conservation Value (F = 0.16 P = 0.69), or Information Availability modules (F = 0.08, P = 0.78). Thus we are confident that assessor bias did not likely influence the remaining assessments that relied on a single assessor.

### Species Doomed to Extinction

Several species merit special mention because of their exceedingly high extinction risk as identified by our expert panels. Because our list was biased initially towards those species exposed to SLR, most but not all of these species are primarily threatened by SLR. We list some examples of these species and the primary factors influencing their extinction risk in Table 1. 

**Table 1:** Examples of species highly likely to be extinct by 2100 under 2 m or less of SLR plus synergistic threats; the Vulnerability (VU) score from SIVVA assessments and primary threats identified by experts are included.

Species	Common name	SIVVA VU Score	Primary Threats
*Ammodramus savannarum floridanus*	Florida Grasshopper Sparrow	0.72	Habitat loss, potentially invasive fire ants and/or disease.
*Cyclargus thomasi bethunebakeri*	Miami Blue	0.94	Habitat loss, mosquito control, SLR.
*Ephyriades brunnea floridensis*	Florida Duskywing	0.95	SLR and barriers to migration.
*Hesperapis oraria*	Gulf Coast Solitary Bee	0.93	Small range, SLR.
*Odocoileus virginianus clavium*	Key Deer	0.86	SLR and barriers to dispersal, genetic swamping or competition with mainland deer if moved to mainland.
*Orthalicus reses nesodryas*	Florida Keys Tree Snail	0.91	Habitat loss to development, SLR.
*Pilosocereus robinii*	Key tree Cactus	0.91	Collection, habitat loss, SLR and storm surge.
*Strymon acis bartrami*	Bartram's Scrub-Hairstreak	0.91	Invasive ants, small range, habitat degradation.
*Sylvilagus palustris hefneri*	Lower Keys Rabbit	0.90	Lack of freshwater, SLR, barriers to dispersal.
*Diadophis punctatus acricus*	Key Ringneck Snake	0.91	SLR and barriers to dispersal, genetic swamping with mainland subspecies if moved.

### Uncertainty and Missing Information

The SIVVA module scores vary from zero to one, where one is the maximum attributable priority. We considered species with a combined Vulnerability and Lack of Adaptive Capacity uncertainty that encompassed 1/3 of this scale to have too much uncertainty for prioritization. Surprisingly, only 15/300 species met this criterion. The mean level of uncertainty for each module in SIVVA was +/- 0.16 for Vulnerability, 0.19 for Lack of Adaptive Capacity, and 0.05 for Conservation Value. Experts displayed zero uncertainty regarding the published data available for these taxa. The greatest and most consistent uncertainties corresponded to the distributions of some rare coastal invertebrates, the responses of fishes to SLR, and reproductive rates of many invertebrate taxa. The criteria most commonly ranked as uncertain were the response of species to changes in overall and seasonal rainfall (marked as uncertain for 38% and 44% of species, respectively), response to projected changes in temperature (41%), the impacts of changes in biotic interactions (62%), and vulnerability to synergistic impacts of known threats (54%). Experts also consistently noted uncertainty in the adaptive capacity of species relative to phenotypic plasticity (44%) and the ability of species to recruit to novel habitats (61%). Expert uncertainty regarding the conservation value of species was greatest for the degree to which the species plays a keystone or foundation role in its environment (33%), and the probability of recovery success (58%). 

**Figure 1:**
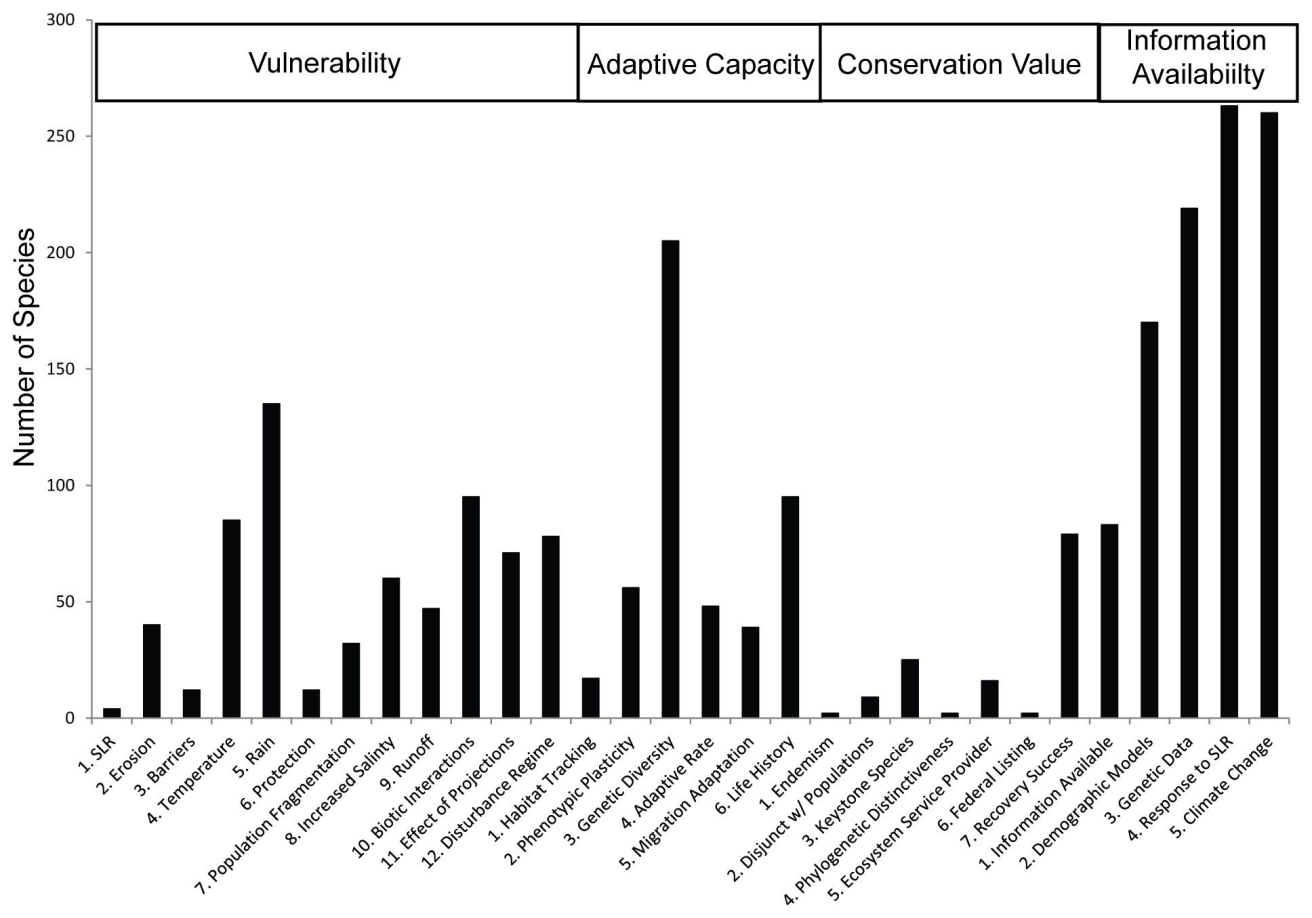
Number of species (out of 300 total) with missing information for all criteria within each of the four SIVVA modules. Criteria were counted as missing when experts choose “0”, or “not enough information to assess”, except for criteria within the Information Availability module, in which case missing information correspond to the assessor choosing “1”, or “no published or unpublished data available.” The types of information most commonly missing were genetic information, basic life history data, and the response of species to projected changes in precipitation. Most species lacked published data on observed or modeled responses to climate change or sea level rise.

 On average, our experts scored 81% of the 12 Vulnerability criteria, 74% of the six Lack of Adaptive Capacity criteria, and 94% of the seven Conservation Value criteria; all Information Availability criteria were scored. The percentages of criteria answered by taxonomic groups are presented in Table 2. Invertebrates consistently displayed the lowest response level in terms of criteria addressed, and mammals consistently displayed the highest response levels. The total number of assessments missing data for each criterion is presented in Figure 1. 

**Table 2:** Percentages of criteria evaluated by experts for taxonomic groups and by each SIVVA module: Vulnerability (VU), Lack of Adaptive Capacity (LAC), and Conservation Value (CV); all Information Availability criteria were evaluated; the mean response level (including Information Availability), weighted by the number of criteria in each module, is also provided.

	VU	LAC	CV	Mean
Birds	92%	80%	95%	90%
Invertebrates	54%	48%	79%	60%
Mammals	94%	91%	100%	95%
Plants	87%	79%	98%	88%
Fishes	81%	71%	94%	82%
Reptiles	90%	90%	100%	93%
Amphibians	96%	83%	100%	94%

### Range of Values for Each Module

**Figure 2:**
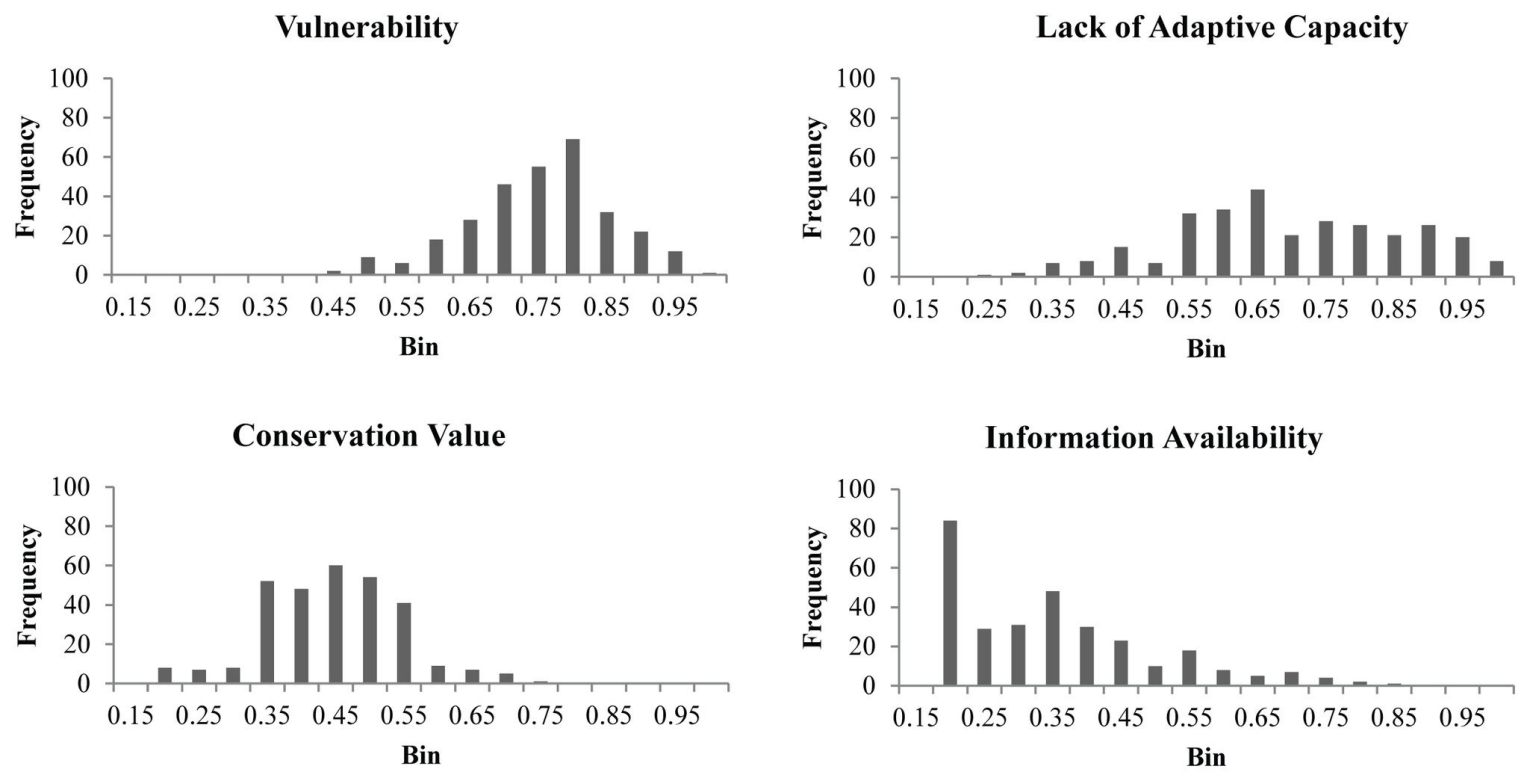
Histograms of SIVVA scores for all 300 species. The number of species in each bin (frequency) is given. Histograms depict the range and dispersion of values for the 300 species surveyed. SIVVA scores follow a statistically normal distribution for Vulnerability, a dispersed distribution for Lack of Adaptive Capacity, a clustered distribution for Conservation Value, and a high frequency of species with low scores for Information Availability, and very few species with high scores for this module.

The average SIVVA scores for each module, averaged across all four SLR scenarios, were 0.73 for Vulnerability, 0.67 for Lack of Adaptive Capacity, 0.42 for Conservation Value, and 0.32 for Information Availability. Given that a score of 0.5 corresponds to zero vulnerability (scores between 0 and 0.5 correspond to positive effects of climate change, SLR, urban encroachment, etc.), it is not surprising that our list of species (all of which are already of conservation concern) displayed relatively high levels of Vulnerability and low Adaptive Capacity (high scores in this module correspond to low vital rates, etc.). Contrastingly, Conservation Value and Information Availability scores varied from low value/information (score of zero) to high value/information (score of one). The relatively low conservation values of species on this list may result from the prevalence of plants (39% of total species list) and invertebrates (22%) that are traditionally not considered to be of high economic, social, or conservation value relative to birds and mammals. We also considered species of greater conservation value when they are phylogenetically distinct, such as a monotypic genus; however, many of the near-coastal species tracked by FNAI are endemic subspecies restricted to barrier islands or the Florida Keys, and generally do not represent long, unique evolutionary histories. The scores follow a bell-curve distribution for Vulnerability, a dispersed or flat distribution for Lack of Adaptive Capacity, are clustered in the mid-range for Conservation Value, and steadily decrease in frequency from low to high scores for Information Availability (Figure 2). 

### Significant Differences between Taxonomic Groups in VU, LAC, CV, and IA

The distribution of Vulnerability, Lack of Adaptive Capacity, Conservation Value, and Information Availability also showed strong taxonomic biases (Table 3). Amphibians and fishes showed the lowest mean Vulnerability scores, while reptiles and invertebrates showed relatively high Vulnerabilities. Plants tended to have the lowest Adaptive Capacity for threats from climate change, land use, and SLR. Conservation Values were fairly even, but highest among mammals and reptiles. Information Availability was dramatically lower for invertebrates and highest for birds and mammals. Table 4 displays pairwise comparisons of mean scores for each module across all taxonomic groups.

**Table 3:** Mean SIVVA scores on a scale from zero (low vulnerability or priority) to one (high vulnerability or priority), values are given for each of the four SIVVA modules corresponding to Vulnerability (VU), Lack of Adaptive Capacity (LAC), Conservation Value (CV), and Information Availability (IA).

Taxonomic Group	VU	LAC	CV	IA
Birds	0.72	0.58	0.40	0.46
Invertebrates	0.77	0.66	0.41	0.25
Mammals	0.71	0.62	0.48	0.51
Plants	0.73	0.75	0.41	0.24
Fishes	0.56	0.52	0.37	0.37
Reptiles	0.76	0.66	0.48	0.43
Amphibians	0.60	0.62	0.40	0.44

Significant comparisons between taxonomic groups for each module’s score are given in Table 4.

**Table 4:** Results of pairwise t-tests comparing taxonomic groups for SIVVA scores in Vulnerability (below diagonal in top panel), Lack of Adaptive Capacity (above diagonal in top panel), Conservation Value (below diagonal in lower panel), and Information Availability (above diagonal in lower panel); asterisks indicate significant differences according to t-tests assuming unequal variance with a BH [69] correction for multiple comparisons.

	Birds	Inverts	Mammals	Plants	Fishes	Reptiles	Amphibs.
Birds		*		*		*	
Inverts	*			*	*		
Mammals		*		*	*		
Plants		*			*	*	*
Fishes	*	*	*	*		*	*
Reptiles					*		
Amphibs.	*	*	*	*		*	
	Birds	Inverts	Mammals	Plants	Fishes	Reptiles	Amphibs.
Birds		*		*	*		
Inverts			*		*	*	*
Mammals	*	*		*	*		
Plants			*		*	*	*
Fishes			*				
Reptiles	*	*		*	*		
Amphibs.			*			*	

### SIVVA Scores between Listed and Non-Listed Species

Species that were federally or state listed as threatened or endangered did not show significantly different Vulnerability scores (P = 0.58, t = 1.97, df = 229). The Lack of Adaptive Capacity scores of listed species was significantly higher (i.e., less adaptive capacity; P < 0.001, t = 1.97, df = 271). Listed species displayed greater Conservation Value (P < 0.001, t = 1.97, df = 267), which is not surprising because listing status was a component of this SIVVA module. Lastly, the SIVVA metric for Information Availability was not significantly different for listed species versus non-listed species (P = 0.38, t = 1.97, df = 286). 

 NatureServe Conservation Status Ranks [43] characterize extinction risk from high to low according to a numerical scale from 1 to 5 (1 = high risk, 5 = low risk). Vulnerability and Lack of Adaptive Capacity scores were reflective of NatureServe State Ranks (S1-S5, as compared to Global Ranks of G1-G5). Species ranked S1 displayed mean SIVVA scores (for Vulnerability and Lack of Adaptive Capacity combined) of 0.74; S2-ranked species had mean scores of 0.68; S3 were 0.67, and S4 were 0.53. These results suggest that the first two modules of SIVVA reflect conservation priorities based on NatureServe State Ranks. 

### Prioritization Lists Overall and For Each Taxonomic Group

**Figure 3:**
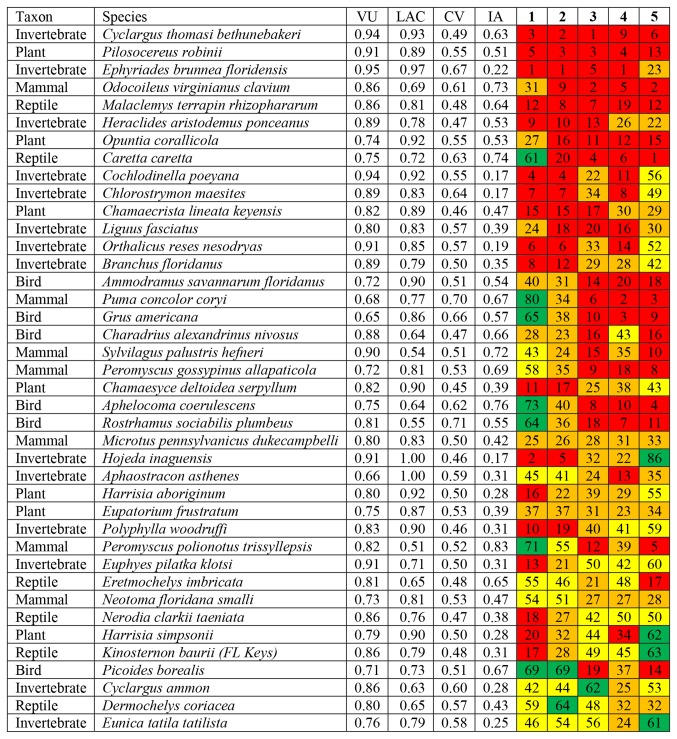
Forty species consistently ranked as having the highest combined vulnerability to threats (VU), lack of adaptive capacity (LAC), conservation value (CV), and information availability (IA). Five weightings schemes are presented, corresponding to 1: stepwise (see methods), 2: 45/25/20/10 percentage weighted averaging of scores for VU, LAC, CV, and IA, respectively, 3: 25/25/25/25 weighting, 4: 20/20/50/10 weighting, and 5: 15/15/35/35 weighting. Species are sorted by the average rank across all five weighting schemes, ranging from 1^st^ to 86^th^ rank (where 1 indicates the highest conservation priority). Red denotes species ranked in the top quartile of this range, orange in the second quartile, yellow in the third, and green in the fourth. The number within each colored square is the relative rank of that species under that weighting scheme. Note that some species consistently fall within the high priority (top) quartile, while others vary depending on what type of information is emphasized in a given ranking scheme.

We prioritized species for conservation efforts by calculating a summary SIVVA score across all four SLR scenarios, generally giving highest priority to those species with the greatest vulnerability to threats, the lowest adaptive capacity, the greatest conservation value, and the greatest information availability for making species-specific conservation decisions. Nevertheless, these factors may be weighted in many ways, so we calculated a SIVVA score under five weighting schemes. The relative rank of each species under each scheme, and the average rank across all schemes, can be viewed in Table S2. Based on the average rank across all five schemes, the top ten species most highly-ranked for conservation efforts are as follows (in order of priority): The Miami blue butterfly (*Cyclargus thomasi bethunebakeri*), Key tree cactus (*Pilosocereus robinii*), Florida duskywing (*Ephyriades brunnea floridensis*), Key deer (*Odocoileus virginianus clavium*), mangrove terrapin (*Malaclemys terrapin rhizophararum*), Schaus swallowtail (*Heraclides aristodemus ponceanus*), Florida semaphore cactus (*Opuntia corallicola*), loggerhead turtle (*Caretta caretta*), truncate urocoptid (*Cochlodinella poeyana*), amethyst hairstreak (*Chlorostrymon maesites*), and narrowpod sensitive pea (*Chamaecrsrista lineata keyensis*). The forty species with the highest average scores across all prioritization approaches are given in Figure 3. The three top-priority species for each taxonomic group are listed in Table 5.

**Table 5:** Three highest ranked species for each taxonomic group; species were identified as having the highest priority across all modules, averaged across all four SLR scenarios based on their mean rank across all five prioritization schemes and out of 300 total species (1 being the highest conservation priority).

Taxon	Species	Average Rank
Birds	*Ammodramus savannarum floridanus*	25
	*Grus americana*	25
	*Charadrius alexandrinus nivosus*	25
Invertebrates	*Cyclargus thomasi bethunebakeri*	4
	*Ephyriades brunnea floridensis*	6
	*Heraclides aristodemus ponceanus*	16
Mammals	*Odocoileus virginianus clavium*	10
	*Puma concolor coryi*	25
	*Sylvilagus palustris hefneri*	25
Plants	*Pilosocereus robinii*	6
	*Opuntia corallicola*	16
	*Chamaecrista lineata keyensis*	21
Fishes	*Etheostoma okaloosae*	73
	*Rivulus marmoratus*	136
	*Menidia conchorum*	165
Reptiles	*Malaclemys terrapin rhizophararum*	12
	*Caretta caretta*	18
	*Eretmochelys imbricata*	37
Amphibians	*Notophthalmus perstriatus*	90
	*Rana okaloosae*	106
	*Ambystoma cingulatum*	231

## Discussion

We present the first statewide combined vulnerability assessment and conservation prioritization that includes multiple threats to species, including SLR, climate change, and land-use intensification, as well as other factors such as adaptive capacity; ecological, evolutionary, economic and social value; and the amount of life history, genetic, and population information available for crafting meaningful conservation plans or actions. We prioritize 300 species by these factors and provide rankings for each species under five different value schemes. Our prioritization system, the Standardized Index of Vulnerability and Value Assessment (SIVVA), also provides a mechanism for exploring the relative rank of each species across different value or weighting schemes, emphasizing for example, vulnerability over conservation value or vice versa. 

### Comparison with Other Vulnerability Assessments

Several systems for assessing vulnerability and conservation priority currently exist, with the most commonly used including the US and state-level threatened and endangered species lists, NatureServe Conservation Status Assessments (i.e., Global/State status ranks), and the International Union for the Conservation of Nature (IUCN) Red List. However, the same species often receive highly disparate ranks across these systems [34], and in most cases the reason for different valuations of the same species are not transparent. Whereas the ESA, IUCN, and NatureServe approaches did not historically account for climate change or SLR directly, some assessments do, such as the Climate Change Vulnerability Index (CCVI), and recent changes to the IUCN approach also include climate change. We elected against using the CCVI for this study because 1) it only characterizes vulnerability and ignores population trends and other important facets of prioritization such as ecological [44], evolutionary [45,46], and economic value [18], and the likelihood of conservation success [18,47,48]; 2) the CCVI uses masked functions for weighting different types of information; 3) these hidden value schemes weight changes from temperature and precipitation as being twice as important as SLR in the computing of overall vulnerability, which seemed to us arbitrary and inflexible. Our assessment, SIVVA, incorporates most of the criteria in existing assessments (Table S1) [34] and provides a clear weighting scheme for each of 30 criteria across four different types of information (Vulnerability, Lack of Adaptive Capacity, Conservation Value, and Information Availability). 

 Our assessments depended on informed expert opinion. Although most assessments were completed by individuals or small groups of individuals working together, a subset of 40 species was evaluated by two independent groups, and we show that assessor bias was not a significant source of variation among scores. This consistency results from several efforts: 1) By providing experts with thorough guidance on our assessment, we avoided interpretation bias. 2) Clear explanation of scoring criteria for each question and generalized categories for scoring reduced ambiguity [47]. For example, experts were asked to choose the highest value possible (our scoring scheme varied from zero to six) for vulnerability to SLR when projections inundated “50 to 100% of known range,” a generalization that helps to avoid assessor disagreement over the difference, for example, between 60% versus 70% inundation, but still distinguishes such species from those with less than 50% inundation. 3) Experts were provided with a literature review for each species; relying on expert opinion in combination with peer-reviewed literature has been shown to greatly improve expert-based assessments [37,49]. 

 Multiple types of uncertainty exist in vulnerability assessments [50], including uncertainty expressed by the experts, uncertainty introduced by a lack of available information, and uncertainty or variation due to how different types of information are weighted relative to each other, also referred to as “hidden value judgments” [47]. Our assessments in SIVVA quantitatively account for all three types of uncertainty and represent an improvement over existing methods that either ignore or fail to quantify uncertainty (e.g., US ESA, IUCN), or only address uncertainty expressed by the expert assessors (e.g., CCVI) or geographic uncertainty in exposure to threats (Conservation Status Assessment). 

### Taxonomic Patterns of Vulnerability, Value, and Missing Data

Conservation science is known to have a strong taxonomic bias towards mammals and birds and against plants, amphibians, and especially invertebrates and microbes [51-54]. Compared to previous assessments [52], the taxonomic breakdown of our species lists closely reflects the overall distribution of taxa (except microbes), though perhaps over-representing plants and under-representing fishes and amphibians. In addition to making up a relatively small proportion of species sampled, fishes and amphibians showed significantly lower Vulnerability scores than all other groups (Table 4), and the lowest Conservation Value scores, on average, relative to other taxonomic groups. The low conservation value estimates for fish and amphibians are common in both the ecological [55] and conservation literature [52], yet typically these species are at high extinction risk [56,57]. This discrepancy likely results from the limitation of our search to amphibian species with high exposure to SLR, which did not include the many imperiled inland amphibian taxa. Fishes and invertebrate species also showed the lowest levels of Information Availability in terms of publications, basic life history data, and studies or models of responses to climate change and SLR. These biases in information availability are similar to those reported by others [51,58] and demonstrate a need for greater taxonomic breadth of research. All taxonomic groups showed a lack of published information on genetic variability and responses to climate change and SLR (Figure 1).

 Several species, in particular invertebrates, were ranked as at high risk of extinction, but do not receive high priority for conservation due to a lack of basic life history information. Examples include the Keys scaly cricket (*Cycloptilum irregularis*), the mangrove long-horned beetle (*Heterachthes sablensis*), and the Antillean spreadwing (*Lestes spumarius*). We do not advocate abandoning these and similar species. Nevertheless, conservation actions that target species or groups of species should, whenever possible, be based on knowledge of the life history and ecology of target species [59] because of the well-established individualistic responses of species to environmental change [60]. Without life-history information that would indicate potential responses to alternative management actions, we would not give high priority for conservation action to these species, aside from protecting known occurrences. On the other hand, they should receive high priority for basic research. Thus, for many species, additional research must be conducted before conservation measures beyond protecting documented populations can be successfully implemented [61].

 Invertebrates make up 35% of the 40 highest priority species (Figure 3), despite constituting 22% of all species evaluated, and no fish or amphibian species make this list, due to their low representation in our full list of 300 species. Birds make up approximately 15% of both the full and the high priority list, and mammals 17% of high priority species, but only 9% of the full list of 300 species assessed. Our high priority list also under-represents plants (18% versus 39%); this likely reflects the numerous varieties and subspecies of plants tracked by FNAI, which receive low conservation value in terms of evolutionary uniqueness. The benefit of our approach is that while taxonomic biases may exist in resulting prioritizations, they have a traceable empirical basis. Given the taxonomic biases in our initial list of 300 taxa and in our final prioritizations, we also provide prioritized lists of species by taxonomic group (Table 5) and their mean priority level in the assessment of all species. We suggest that these results be incorporated into state wildlife action plans and similar efforts as a way of prioritizing the listing of species, conservation and recovery efforts, and potentially funding, although equal priority does not necessarily equate to equal funding needs because some species are inherently more costly to conserve than others [62].

### Prioritization List and Conservation Recommendations

Adopting an approach such as SIVVA would make endangered species listing and conservation/recovery planning more transparent, rational, and empirical, an improvement that is sorely needed [18,47]. We show that, at present, listed species in Florida (at the state and/or federal level) do not consistently show higher vulnerabilities to current or future threats than non-listed species, suggesting that prioritizing conservation efforts by listing status may not protect the species in greatest need of conservation interventions. Prioritizations under SIVVA do reflect NatureServe Conservation Status Assessment ranks, but only in terms of vulnerabilities to threats, adaptive capacity, and some components of conservation value, such as endemism (narrow geographic distribution). Because other factors such as ecological, evolutionary, economic, and social values attributed to species should also factor into conservation prioritization, using an approach such as SIVVA could better synthesize these disparate types of information. 

Our experts identified several species (Table 1) as virtually certain to be extinct or functionally extinct [63] by 2060, given current population trends and vulnerability to projected threats from SLR, climate change, and land-use change. Nevertheless, not all of these species are top priorities for conservation efforts in our final analyses (Figure 3). Species highly vulnerable to extinction should receive high priority for conservation efforts under a strategy to “prevent all extinctions,” but this is not the only value system in conservation biology. For example, many argue that in addition to extinction vulnerability, conservation efforts should be directed towards species that provide the greatest benefits to humans [64-66] or to other species or ecosystems [44,67,68], or that have a high likelihood of cost-effective recovery [18]. Given that conservation practitioners commonly disagree on priorities, prioritization systems should be flexible enough for users with different value systems to use the same underlying data. Our implementation of SIVVA does exactly this; moreover, as shown in Figure 3, it allows visualization of the relative priority of species and their mean priority across multiple competing value systems. Florida’s land-management and conservation agencies could revise these findings under their own value systems and prioritize taxa by placing greater weight, for example, on ecological or economic value, or focus entirely on vulnerability and ignore conservation values. A major benefit of a system such as SIVVA is that it would make the allocation of conservation effort more quantitative and transparent. 

## Supporting Information

Table S1 **SIVVA’s four criteria categories (referred to as “modules” in text), the weight or importance of each criterion in our assessment, and the criteria within each module.** X's denote presence of the criteria in existing vulnerability assessments including the Climate Change Vulnerability Index (CCVI), International Union for the Conservation of Nature Red List (IUCN), NatureServe Conservation Status Assessment (CSA), and US Endangered Species Act (US ESA).
(DOCX)

Table S2 **Latin names of species evaluated, organized by taxonomic group.** Species names in bold comprise 32 species added to initial sea level rise analysis (see Methods).
(XLSX)

Table S3 **List of taxonomic experts who participated in this study.** In addition to those listed here, expert assessors included those listed by Dubois et al. (2011), and eight anonymous assessors.
(DOCX)
