# Supplementary material for: A Vulnerability Assessment of 300 Species in Florida: Threats from Sea Level Rise, Land Use, and Climate Change
Source: PLoS One. 2013 Nov 19;8(11):e80658. doi: 10.1371/journal.pone.0080658 (PMC3834108; doi:10.1371/journal.pone.0080658)
Supplement: Table S1 — SIVVA’s four criteria categories (referred to as “modules” in text), the weight or importance of each criterion in our assessment, and the criteria within each module. X's denote presence of the criteria in existing vulnerability assessments including the Climate Change Vulnerability Index (CCVI), International Union for the Conservation of Nature Red List (IUCN), NatureServe Conservation Status Assessment (CSA), and US Endangered Species Act (US ESA). (DOCX) [file pone.0080658.s001.docx]

| **Criteria** | | **Weight** | **CCVI** | **IUCN** | **CSA** | **US ESA** |
| --- | --- | --- | --- | --- | --- | --- |
| ***Vulnerability*** | |  |  |  |  |  |
|  | 1. Sea Level Rise | 4 | X |  | X | X |
|  | 2. Erosion | 0.5 |  |  |  | X |
|  | 3. Barriers to Movement | 4 | X |  |  | X |
|  | 4. Temperature | 1 | X |  |  |  |
|  | 5. Precipitation | 1 | X |  |  |  |
|  | 6. Portion of Range Protected | 1 | X |  |  | X |
|  | 7. Population Fragmentation | 1 | X | X | X | X |
|  | 8. Increasing Salinity | 1 |  |  |  | X |
|  | 9. Storm Surge or Run-off | 1 |  |  |  |  |
|  | 10. Biotic Interactions | 2 | X |  | X | X |
|  | 11. Synergistic Threats | 1 |  |  |  |  |
|  | 12. Disturbance Regime | 2 | X |  | X | X |
| ***Adaptive Capacity*** | |  |  |  |  |  |
|  | 13. Migration | 2 | X |  |  |  |
|  | 14. Phenotypic Plasticity | 2 | X |  |  |  |
|  | 15. Genetic Diversity | 1 | X |  |  |  |
|  | 16. Adaptive Rate | 1 |  | X | X | X |
|  | 17. Demographic Capacity | 1 | X | X | X | X |
|  | 18. Colonization Potential | 1 | X | X |  |  |
| ***Conservation Value*** | |  |  |  |  |  |
|  | 19. Level of Endemism | 2 |  | X |  |  |
|  | 20. Disjunct Population | 1 |  |  |  |  |
|  | 21. Keystone Species | 2 |  |  |  | X |
|  | 22. Phylogenetic Distinctiveness | 2 |  |  |  |  |
|  | 23. Economic Value | 1 |  |  |  |  |
|  | 24. State or Federal Listing | 1 |  |  |  | X |
|  | 25. Probability of Recovery | 1 |  |  |  |  |
| ***Information Availability*** | |  |  |  |  |  |
|  | 26. Published Literature | 2 |  |  |  |  |
|  | 27. Demographic/Niche Models | 1 | X | X |  |  |
|  | 28. Population Genetic Studies | 1 |  |  |  |  |
|  | 29. Response to Sea Level Rise | 1 |  |  |  |  |
|  | 30. Response to Climate Change | 1 | X |  |  |  |
