# Supplementary material for: A Vulnerability Assessment of 300 Species in Florida: Threats from Sea Level Rise, Land Use, and Climate Change
Source: PLoS One. 2013 Nov 19;8(11):e80658. doi: 10.1371/journal.pone.0080658 (PMC3834108; doi:10.1371/journal.pone.0080658)
Supplement: Table S3 — List of taxonomic experts who participated in this study. In addition to those listed here, expert assessors included those listed by Dubois et al. (2011), and eight anonymous assessors. (DOCX) [file pone.0080658.s003.docx]

| Assessor | Institution |
| --- | --- |
| Reed Bowman | Archbold Biological Station |
| Natalie Dubois | Defenders of Wildlife |
| Joyce Maschinski | Fairchild Botanical Garden |
| Marianne Korosy | Audubon of Florida |
| GB Edwards | Florida Department of Agriculture |
| Mike Thomas | Florida Department of Agriculture |
| Paul Skelley | Florida Department of Agriculture |
| Jim Wiley | Florida Department of Agriculture |
| Bill Mauffray | Florida Department of Agriculture |
| Beth Stys | Florida Fish and Wildlife Conservation Commission |
| Bob Glazer | Florida Fish and Wildlife Conservation Commission |
| John Slapcinski | Florida Museum of Natural History |
| Amy Jenkins | Florida Natural Areas Inventory |
| Kim Gulledge | Florida Natural Areas Inventory |
| Ann Johnson | Florida Natural Areas Inventory |
| Dale Jackson | Florida Natural Areas Inventory |
| Dan Hipes | Florida Natural Areas Inventory |
| Dave Almquist | Florida Natural Areas Inventory |
| Kathleen NeSmith | Florida Natural Areas Inventory |
| Jon Oetting | Florida Natural Areas Inventory |
| Keith Bradley | Institute for Regional Conservation |
| George Gann | Institute for Regional Conservation |
| Andrew Warren | McGuire Lepidopteran Center |
| Angela Tursi | University of Central Florida |
| Reed Noss | University of Central Florida |
| Christopher Parkinson | University of Central Florida |
| Eric Hoffman | University of Central Florida |
| Jack Stout | University of Central Florida |
| Gregory Territo | University of Central Florida |
| Michael Volk | University of Florida |
| Tom Hoctor | University of Florida |
| James Watling | University of Florida |
| Allison Benscoeter | University of Florida |
| Jodie Smithem | US Fish and Wildlife Service |
| Laura Brandt | US Fish and Wildlife Service |
| Steve Traxler | US Fish and Wildlife Service |
| Stephanie Romanach | US Geological Service |
| Joshua Reece | Valdosta State University |
